# Selected Lines and Inbred Strains: Tools in the Hunt for the Genes Involved in Alcoholism

**Published:** 2000

**Authors:** Nicholas J. Grahame

**Affiliations:** Nicholas J. Grahame, Ph.D., is an assistant scientist in the Department of Medicine, Indiana University School of Medicine, Indianapolis, Indiana

**Keywords:** animal model, animal strains, animal selectively bred for AOD (alcohol or other drug) preference, selective breeding, genetic theory of AODU (AOD use, abuse, and dependence), phenotype, quantitative trait locus, gene knockout technology, transgenic technology

## Abstract

In their quest to elucidate the genetic influences contributing to alcoholism, researchers have long used selected lines and inbred strains of rodents. Selected lines are obtained by repeatedly mating those animals within a population that show extremely high or low values of the desired trait. Inbred strains are generated by mating male and female siblings, irrespective of any particular trait, over several generations. Both of these approaches have provided researchers with extensive knowledge about the genetic and neurobiological mechanisms contributing to alcohol-related traits. However, the use of these models is associated with some limitations, mostly resulting from the inbreeding involved in generating such lines and strains. Nevertheless, these models can offer some advantages over other genetic approaches, such as the analysis of quantitative trait loci or the generation of transgenic and knockout mice.

Breeding techniques to generate animals with desired traits have long been a staple of genetic research, including alcohol-related research. Two of the oldest techniques for studying the genetics of alcohol-related traits in animals are analyses of selected lines and of inbred strains, usually of rats or mice. The use of these animal models long predates the present revolution in molecular biology, because it does not necessitate advanced biological techniques. Like newer models using molecular genetic techniques, however, these breeding approaches are based on the concept of manipulating an animal’s genetic material and studying the resulting effects on a behavior of interest.

Alcohol studies using selected lines and inbred strains rely on the study of groups, or populations, of animals that differ on a genetic level. At the same time, the animals’ environment can be controlled rigorously in the laboratory setting. Under these conditions, comparisons of various populations allow alcohol researchers to investigate how genes can influence a wide variety of alcohol-related behaviors (e.g., alcohol consumption, tolerance, and withdrawal) as well as physiological traits that may be important in mediating alcohol’s effects. This article briefly reviews the strategies used in generating selected lines and inbred strains. The article then discusses some of the applications of these models as well as some of the limitations associated with their use.

## Generation of Selected Lines

Selective breeding has long been used in agriculture to enhance desired characteristics (i.e., phenotypes) in both plants and animals. In the laboratory, researchers create selected lines by exploiting the natural variability inherent in an animal population and breeding those individuals that have either extremely high or extremely low values of the phenotype of interest. For example, in regard to alcohol withdrawal, researchers might mate either animals exhibiting the most severe withdrawal symptoms or animals exhibiting the mildest withdrawal symptoms. After several generations of selective breeding, the resulting lines will demonstrate stable differences in the phenotype of interest.

The most widely studied alcohol-related phenotype in selected lines has been free-choice alcohol drinking. Although many animals will not drink alcohol when given a choice between alcohol and water, great variability exists in the amount of alcohol individual animals within a population will consume. Selective breeding for differences in this phenotype was first initiated in the late 1940s, when Mardones and colleagues developed the UChA and UChB lines of rats that exhibit low and high levels of alcohol consumption, respectively (Mardones and Segovia-Riquelme 1983). Since then, researchers have generated numerous sets of selected lines of rats and mice that differ in free-choice alcohol intake.

As with the molecular biological approaches discussed in other articles in this journal issue, selected lines result from the experimental manipulation of genes that cause differences in a phenotype. When selection occurs toward both high and low levels of the phenotype of interest (called bi-directional selection), the two lines will show progressively greater differences (i.e., divergence) throughout the generations with respect to the trait of interest and in the genes underlying this trait. With respect to other traits and their underlying genes, however, the two lines should remain similar. Thus, in the case of free-choice alcohol consumption, selective breeding results in two lines that have different variants (i.e., alleles) of the genes related to drinking but similar alleles of genes unrelated to drinking (e.g., coat color).^1^

## Generation of Inbred Strains

In contrast with selected lines, in which unrelated animals with similar characteristics are mated, or crossed, inbred strains are created by crossing male and female siblings. The offspring of this cross again are mated to each other and so on for 20 consecutive generations. The result of this procedure is a population of animals in which only one allele of every gene is present (i.e., which is homozygous for every allele). Consequently, all animals within an inbred strain are genetically identical, akin to identical twins. Researchers can reliably study these populations, because their genetic makeup has been effectively fixed and will not change, except for changes resulting from spontaneous alterations (i.e., mutations) in an individual’s genetic material. Any differences that exist between individual members of a strain most likely can be attributed to environmental influences.

To study the role of the genes underlying the trait of interest, researchers must assess and compare numerous inbred strains. Differences between different inbred strains having the same environmental history can be attributed to genetic differences. Alcohol researchers have used this approach since the late 1950s, when McClearn and Rodgers (1959) published seminal articles demonstrating differences in free-choice alcohol consumption among numerous inbred strains of mice. In those studies, the level of free-choice alcohol consumption differed over tenfold among the inbred strains tested. Furthermore, because the animals were raised under identical environmental conditions, these differences must have resulted from genetic differences between the strains. Unlike selected lines, however, which should differ from each other only with respect to the traits related to the behavior under investigation, inbred strains will differ from each other on a wide variety of traits both related and unrelated to the behavior under investigation. For example, whereas selected lines bred for high and low alcohol consumption should differ in the amount of alcohol ingested but not in an unrelated trait (e.g., coat color), inbred lines may differ in both alcohol consumption and the unrelated trait.

## Applications for Selected Lines and Inbred Strains in Alcohol Research

Selected lines and inbred strains have provided two important contributions to studies exploring the genetics of alcohol-related traits. First, these animal models have yielded extensive knowledge about the genetic underpinnings of individual differences in alcohol-related traits. For example, these lines and strains have enabled researchers to formulate extensive theories on the causes of high alcohol intake in animals (e.g., Li et al. 1993). In principle, selected lines and inbred strains can be used to determine the genetic factors that are correlated with differences in any alcohol-related behavior of interest as well as the effects of chance differences between the lines and strains (Crabbe et al. 1990; Falconer and Mackay 1996).

The systematic differences between animal strains or individual animals with respect to an alcohol-related trait (e.g., alcohol consumption level) frequently are caused by genes that affect multiple phenotypes (i.e., have pleiotropic effects). Such pleiotropic effects may hint at the causes underlying a certain target trait. For example, both in selected lines and inbred strains of mice a highly consistent, negative genetic correlation exists between free-choice alcohol consumption and alcohol withdrawal. This means that selected lines and inbred strains which experience comparatively mild alcohol withdrawal also drink more alcohol and vice versa (Metten et al. 1998). Such a correlation indicates that the genes influencing the severity of alcohol withdrawal also affect alcohol consumption and implies that alcohol withdrawal discourages voluntary alcohol drinking in mice.

Analyses of the genetic differences between selected lines exhibiting high and low alcohol consumption also have helped researchers assess neurobiological differences between selected lines. Such studies have found consistent innate differences between high- and low-consuming selected lines (McBride and Li 1998). For example, some animals from high-preferring lines that have never been exposed to alcohol (i.e., alcohol-naive animals) show lower levels of the brain chemical (i.e., neurotransmitter) serotonin than do alcohol-naive animals from low-preferring lines. This type of analysis enables scientists to identify potential mechanisms underlying alcohol consumption and to distinguish those mechanisms from the effects of alcohol consumption on brain function. Scientists would have difficulty conducting these analyses in humans, because controlling for their alcohol-drinking history is impossible. When interpreting the results of such studies, however, one must always consider that free-choice alcohol consumption (or any alcohol-related phenotype) in rodents is a model for the human condition that likely reflects some but not all of the elements contributing to human alcohol use and alcoholism.

The second important contribution of inbred strains and selected lines to alcohol research has been that these animal models can consistently exhibit a phenotype otherwise considered rare in the “outbred,” or nonselected, animals commonly used in laboratories. For example, high free-choice alcohol consumption is an uncommon behavior in most rodents; accordingly, studies on the effects of alcohol consumption would have to involve many animals, most of which could not be used because they do not show the desired behavior. Certain inbred strains (e.g., C57/BL6 mice or Fawn-Hooded rats), however, require little training to initiate alcohol consumption (George 1987), as do selectively bred rats or mice (Froehlich 1995; Grahame et al. 1999). A large proportion of those animals will exhibit the trait of interest, allowing researchers to perform experiments (e.g., assessing the environmental and physiological factors that affect alcohol intake or testing medications designed to reduce drinking) without having to eliminate many animals that do not meet the alcohol consumption criteria.

### Potential Caveats of Animal Models

In addition to the previously mentioned fact that animal models likely provide only an incomplete representation of human behaviors as complex as alcohol use and abuse, several other potential problems exist in interpreting the findings of such research. Crabbe and colleagues (1990) have examined extensively the caveats associated with experiments assessing genetic correlations in inbred strains and selected lines. For example, researchers must consider several factors when trying to determine whether the correlation between two traits (e.g., alcohol consumption and alcohol withdrawal) actually arises from the pleiotropic actions of the gene or genes that underlie both traits rather than from the actions of two unrelated genes.

The most important of these factors is that to the extent possible, investigators must ensure that differences among several selected lines or inbred strains are not caused by random differences resulting from inbreeding. After repeated inbreeding (which occurs in both selected lines and inbred strains), both alleles for many genes become fixed within a population—that is, all individuals in that population carry the same allele of a certain gene. This fixation occurs both for genes that are relevant to the trait under investigation (e.g., alcohol consumption) and for genes that are irrelevant to that trait. For example, among inbred mice, animals of the strain C57BL/6 (which readily drink alcohol) have a black coat, whereas animals of the strain DBA/2 (which avoid alcohol) have a tan coat. Thus, one could conclude that the same genes that determine coat color also determine alcohol consumption levels. To support such a conclusion, however, scientists must determine whether a consistent correlation between coat color and alcohol consumption exists in other inbred strains as well. In fact, researchers typically must assess about 12 to 15 inbred strains before the data have sufficient statistical power to detect a robust correlation between two phenotypes (e.g., alcohol consumption and alcohol withdrawal). Such a robust correlation, which is represented by a correlation coefficient r = 0.5–0.6, would mean that approximately 25 percent of the variance observed in alcohol consumption resulted from variance in the other phenotype (i.e., withdrawal).

Studies involving inbred strains therefore require a relatively large number of strains to detect moderate genetic correlations. The analysis of numerous strains decreases the likelihood that differences between strains are caused by random fixation of alleles and increases the likelihood that the results also apply to other strains or organisms (i.e., can be generalized)—an important issue when one hopes to apply the results to humans. Although such an analysis of numerous strains is labor- and cost-intensive, it is the only way to identify correlated traits and calculate a corresponding correlation coefficient.

The potential negative consequences of inbreeding can affect not only inbred strains but also selected lines. Although selective breeding usually specifically avoids mating brothers and sisters, inbreeding still occurs, because the population used for creating a selected line (e.g., those animals showing the highest free-choice alcohol consumption) often is relatively small. In general, the smaller the population is in which selective breeding is performed and the longer selective breeding is carried out, the greater is the level of inbreeding (Falconer and Mackay 1996). This inbreeding can result in the generation of random differences (i.e., genetic drift) between two selected lines. For example, if some of the animals used for generating a high alcohol-consuming selected line by chance have a somewhat lighter coat color than the animals in the original population, the inbreeding inherent in generating the selected line may result in a population with a lighter coat color, even though coat color is unrelated to alcohol consumption. The emergence of such differences in traits unrelated to the phenotype of interest can greatly complicate the identification of genes that help determine the phenotype of interest (for a more mathematical presentation of this issue, see Belknap et al. 1997).

Thus, to maximize the strength of selective breeding—that is, to cause systematic differences related to the selection phenotype to emerge and to minimize differences unrelated to the selection phenotype—experiments involving selected lines should maintain as large a population of breeding animals as is economically feasible. In addition, the generation of replicate selected lines that are bred from different animals to exhibit the same phenotype as the original lines can help researchers interpret differences between selected lines. A correlation that occurs in both the original selected lines and the replicate lines can be considered highly reliable evidence that a true genetic correlation exists rather than a genetic drift (Crabbe et al. 1990).

### Comparison of Selected Lines and Inbred Strains With Other Genetic Approaches

#### Quantitative Trait Loci

Another approach to examining the pleiotropic effects of alleles and dissecting genetic correlations in a particular population involves the identification of quantitative trait loci (QTLs). A QTL is a small section on the cell’s genetic material (i.e., the DNA) that helps shape a quantitative trait—in other words, a phenotype (e.g., alcohol consumption) that is determined by more than one gene, each of which exists in several alleles. Using molecular genetic techniques, researchers can locate such QTLs on the DNA and calculate the magnitude of their contribution to the phenotype under investigation (for more information on QTLs, see the article in this issue by Grisel, pp. 169–174).

Genetic correlation studies using selected lines and inbred strains to identify the genes contributing to a certain phenotype differ in several aspects from the QTL approach. First, QTL studies investigate the influence of a single gene on the phenotype, whereas genetic correlation studies traditionally have been used to detect the influence of a group of anonymous genes that act in concert and which may affect several phenotypes. In other words, selected lines and inbred strains allow researchers to examine the entirety of a genetic correlation and do not attempt to dissect the genetic variance into pieces that are each influenced by one specific gene. This approach improves the statistical power, and thus the replicability, of the analysis, because it does not try to break up genetic sources of variance into discrete units, which can be difficult to detect reliably. The benefits of the genetic correlation approach are particularly great when analyzing phenotypes for which genetic factors play only a moderate role, such as alcohol consumption. For these phenotypes, each QTL may account only for a small portion of the variance in the phenotype, often less than 10 percent.

Conversely, QTL studies may offer some advantages over genetic correlation studies. For example, the failure to detect a genetic correlation using selected lines and inbred strains does not mean that such a correlation does not exist. Instead, the correlation may be highly complex and involve several genes having such diverse (and even opposite) effects that they mask a genetic correlation when they act in concert. In such cases, the QTL approach may enable researchers to find individual alleles that produce such opposite pleiotropic effects. In practice, however, scientists rarely take the trouble to look for QTLs underlying genetic correlations without having initially observed such correlations in animal models. In fact, both selected lines (e.g., Bice et al. 1998) and inbred strains frequently provide a starting point for QTL analyses, because these analyses are easiest when the DNA to be examined is derived from animals with two highly divergent phenotypes.

### Transgenic and Knockout Animals

Two other approaches to examining the function of specific genes and their effects on alcohol-related traits in animal models involve transgenic and knockout mice. In transgenic mice, the gene of interest is altered (i.e., mutated) in a test tube and then introduced into the mice, enabling researchers to study the effects of that gene alteration. In knockout mice, researchers inactivate the gene of interest, allowing them to draw conclusions on the function of that gene by determining the consequences of its absence. (For more information on these animal models, see the article in this issue by Bowers, pp. 175–184). Like the QTL approach, these animal models attempt to analyze complex, alcohol-related phenotypes by investigating the functions of individual genes.

In contrast to selected lines and inbred strains, which take advantage of the genetic variability that occurs naturally in a population, transgenic mice involve the generation of specific mutations in the gene of interest to create new variant alleles. Such targeted mutations are tremendously useful for a trait in which a known mutation exists in humans. This mutation can then be reproduced in transgenic mice in order to study its effects in more detail. In the vast majority of disorders (including alcoholism), however, researchers do not know the allelic variation that leads to differences in human behavior. Therefore, when attempting to model normal variations in humans that lead to differences in alcohol-related behaviors, scientists may prefer to exploit the normal variation present in rodent populations rather than to create artificial genes by generating new mutants.

Furthermore, in contrast with normally occurring alleles found in selected lines and inbred strains, transgenic models using “artificial” alleles may result in phenotypes that are influenced by specific (and often unanticipated) developmental and genetic parameters (for a discussion of this issue with respect to pain research, see Mogil and Grisel 1998). For example, researchers may experience difficulties in replicating even experiments in which the mutations had seemingly large effects, because small changes may have occurred in the DNA near the site of the mutation between the original and the replicate experiments (Phillips et al. 1999). Therefore, although transgenic models are undoubtedly useful when identifying genes involved in physiological functions, researchers should not overlook the importance of normal variation in alleles that is represented by selected lines and inbred strains when attempting to genetically define important alcohol-related phenotypes.

## Summary

Selected lines and inbred strains, both of which rely on the normal genetic variability present within animal populations, continue to be useful tools in understanding the relationship between genes and alcohol-related traits. Much work remains to be done, however, in order to understand how genetic differences lead to behavioral differences in alcohol response. Genetically defined animal models, such as selected lines and inbred strains, will continue to form the basis for this work.

## Footnotes

1 This scenario describes the ideal situation, in which the genes unrelated to drinking are inherited independently of the genes related to alcohol consumption. In reality, some genes often are inherited together (i.e., are linked) even though their functions are unrelated—for example, because the genes are located next to each other on the same chromosome.
